# A Pivotal Role for AP-1-Mediated Osteopontin Expression in the Increased Migration of Vascular Smooth Muscle Cells Stimulated With HMGB1

**DOI:** 10.3389/fphys.2021.775464

**Published:** 2021-11-04

**Authors:** Eun Yeong Jeon, Seung Eun Baek, Ji On Kim, Jong Min Choi, Eun Jeong Jang, Chi Dae Kim

**Affiliations:** ^1^Department of Pharmacology, School of Medicine, Pusan National University, Yangsan, South Korea; ^2^Gene & Cell Therapy Research Center for Vessel-Associated Diseases, Pusan National University, Yangsan, South Korea; ^3^Research Institute for Convergence of Biomedical Science and Technology, Pusan National University Yangsan Hospital, Yangsan, South Korea

**Keywords:** vascular smooth muscle cells, high-mobility group box 1, cell migration, osteopontin, activator protein 1

## Abstract

Migration of vascular smooth muscle cells (VSMCs) plays an essential role in the development of vascular remodeling in the injured vasculatures. Previous studies have identified high-mobility group box 1 (HMGB1) as a principal effector mediating vascular remodeling; however, the mechanisms involved have not been fully elucidated. Thus, this study investigated the role of HMGB1 on VSMC migration and the underlying molecular mechanisms involved. VSMCs were *ex plant* cultured using rat thoracic aorta, and the cellular migration was measured using wound-healing assay. Osteopontin (OPN) mRNA and protein were determined by reverse transcription polymerase chain reaction (RT-PCR) and Western blot, respectively. The OPN promoter was cloned into pGL3 basic to generate a pLuc-OPN-2284 construct. Migration of VSMCs stimulated with HMGB1 (100ng/ml) was markedly increased, which was significantly attenuated in cells pretreated with MPIIIB10 (100–300ng/ml), a neutralizing monoclonal antibody for OPN as well as in cells deficient of OPN. In VSMCs stimulated with HMGB1, OPN mRNA and protein levels were significantly increased in association with an increased promotor activity of OPN gene. Putative-binding sites for activator protein 1 (AP-1) and CCAAT/enhancer-binding protein beta (C/EBPβ) in the indicated promoter region were suggested by TF Search, and the HMGB1-induced expression of OPN was markedly attenuated in cells transfected with siRNA for AP-1. VSMC stimulated with HMGB1 also showed an increased expression of AP-1. Results of this study suggest a pivotal role for AP-1-induced OPN expression in VSMC migration induced by HMGB1. Thus, the AP-1-OPN signaling axis in VSMC might serve as a potential therapeutic target for vascular remodeling in the injured vasculatures.

## Introduction

Increasing evidence shows that abnormal proliferation and migration of vascular smooth muscle cells (VSMCs) are common events in the pathophysiology of many vascular diseases, including atherosclerosis and restenosis after angioplasty (Orr et al., 2010; Yu et al., 2011; Zhou et al., 2016). VSMCs normally reside within the media and remain quiescent in healthy arteries (Zhou et al., 2016); however, VSMCs might be activated in the injured vasculatures. In vascular cells stretched by the endoluminal vascular interventional procedures, VSMCs might be damaged (Zheng et al., 2011) and then release endogenous damage-associated molecular patterns (DAMPs) including high-mobility group box 1 (HMGB1; Land, 2013; Baek et al., 2017).

High levels of extracellular HMGB1 have been detected in human atherosclerotic plaque in blood vessels and are implicated in vascular remodeling by potentiating inflammatory responses (Kalinina et al., 2004; Cai et al., 2015). Reportedly, HMGB1 has been suggested as a key DAMPs mediating the phenotypic modulation of VSMCs induced by interferon-γ (Wang et al., 2017). In response to inflammatory reactions, the contractile phenotype of VSMCs turns into the synthetic phenotype of VSMCs (Shi et al., 2019), which migrate from the media to the intima, leading to intimal hyperplasia and vascular restenosis (Lacolley et al., 2012). Thus, HMGB1 has been implicated as an active player in the development of proliferative vascular diseases *via* phenotypic modulation of VSMCs. However, the precise role of HMGB1 on VSMC migration has not been clarified.

In previous studies, elevated level of osteopontin (OPN) has been demonstrated in human atherosclerotic plaque and neointima after experimental angioplasty (Gluba-Brzózka et al., 2014; Huby et al., 2014). OPN is known as a key player in the development of atherosclerosis and mediates vascular injury responses *via* an increase in extracellular matrix invasion, migration, and proliferation of VSMCs (Li et al., 2007; Qiu et al., 2012; Liu et al., 2014a,b). On the basis of the previous report in which OPN was strongly expressed in the synthetic phenotype of VSMCs (Jiang et al., 2014), OPN has been suggested as a key factor mediating vascular remodeling diseases (Wolak, 2014; Mohamadpour et al., 2015).

Although the vascular remodeling effects of OPN have aroused considerable research interests (de Castro Brás, 2015), the precise molecular mechanisms are unclear. Given the importance of OPN in vascular injury responses, we hypothesized that the OPN signaling axis might mediate VSMC migration induced by HMGB1, a key DAMP mediating cardiovascular injury responses. Thus, this study investigated the active role of OPN on cellular migration using VSMCs cultured from rat thoracic aorta. Moreover, we also clarified the molecular mechanism involved in OPN expression in VSMCs stimulated with HMGB1.

## Materials and Methods

### Ethics Statements and Animals

All animal procedures conformed with the Guide for the Care and Use of Laboratory Animals published by the US National Institute of Health (NIH Publication No.85-23, 2011 revision), and all experimental protocols were approved by the Pusan National University Institutional Animal Care and Use Committee. Sprague–Dawley (SD) rats were purchased from Charles River Breeding Laboratories (Kingston, NY, United States).

### Chemicals and Antibodies

Recombinant human HMGB1 (1690-HMB-050) was purchased from R&D System Inc. (Minneapolis, MN, United States). OPN (ab8448) antibody was purchased from Abcam (Cambridge, MA, United States). Activator protein 1 (AP-1; sc-12632) and β-actin (sc-47778) antibodies were purchased from Santa Cruz Biotechnology Inc. (Beverly, MA, United States). CCAAT/enhancer-binding protein beta (C/EBPβ; 3082S) antibody was purchased from Cell Signaling Technology (Beverly, MA, United States). Horseradish peroxidase (HRP)-conjugated IgG secondary antibody was purchased from Santa Cruz Biotechnology Inc. Thymidine (T9250) was purchased from Sigma-Aldrich (St. Louis, MO, United States).

### Cell Culture

Primary VSMCs were *ex plant* cultured from thoracic aorta of SD rats (7weeks old, male). In brief, rats were euthanized by CO_2_ inhalation and then dissected to separate the thoracic aortas. The excised aortas were cut and explanted in a cell culture dish, containing Dulbecco’s Modified Eagle’s Medium (DMEM; Gibco BRL, Grand Island, NY, United States) with 10% fetal bovine serum (FBS; Gibco BRL). Cells (passages 3–5) were then maintained in DMEM with 10% FBS and antibiotic-antimycotic solution (Gibco BRL) including streptomycin sulfate (0.5–1.5%) and penicillin G (0.5–1.5%) at 37°C.

### Wound-Healing Assay

Wound-healing assay was used to evaluate VSMC migration. In brief, VSMCs were seeded into six-well plates containing DMEM with 10% FBS. The cells were wounded by scraping with a standard 200μl pipette tip to make a gap in the central region of plate. Cellular debris was washed with PBS, and then, cells were incubated with fresh DMEM with 0.5% FBS and 0.1% thymidine. Cell migration was photographed with a microscope (Carl Zeiss, AxioVision Software 200) and measured using ImageJ program.

### Small Interfering RNA Preparation and Transfection

Osteopontin, AP-1, and C/EBPβ siRNA oligonucleotides were synthesized by Bioneer (Daejeon, Korea). Negative control duplex siRNA was used as a control. siRNA molecules were transfected into cells using Lipofectamine 2000 siRNA transfection reagent (Invitrogen, Carlsbad, CA, United States), according to the manufacturer’s instructions. siRNA sequences against OPN, AP-1, and C/EBPβ were as follows: OPN, 5'-GUU CUU UCU GUG CAA GAA A-3' (forward) and 5'-UUU CUU GCA CAG AAA GAA C-3' (reverse); AP-1, 5'-ACU GUA GAU UGC UUC UGU A-3' (forward) and 5'-UAC AGA AGC AAU CUA CAG U-3' (reverse); and C/EBPβ, 5'-GAC AAG CUG AGC GAC GAG U-3' (forward) and 5'-ACU CGU CGC UCA GCU UGU C-3' (reverse).

### Western Blot Analysis

Vascular smooth muscle cells lysates were prepared in ice-cold lysis buffer (Thermo Fisher Scientific, Rockford, IL, United States). Equal amounts of protein were separated on 8–10% polyacrylamide gels under reducing conditions and then transferred into nitrocellulose membranes (Amersham-Pharmacia Biotech, Piscataway, NJ, United States). Membranes were blocked with 5% skim milk in Tris-buffered saline Tween-20 (TBST) for 2h at room temperature and then incubated overnight with primary antibody in 5% skim milk at 4°C. Incubated membranes were then washed with TBST and incubated with HRP-conjugated secondary antibody for 2h at room temperature. Blots were developed using the enhanced chemiluminescence (ECL) Western blotting detection reagents (Amersham-Pharmacia Biotech), and then, blots were captured utilizing image capturing software (Amersham, Imager 680 version. 2.0.). Signal bands were quantified using the UNSCAN-IT CEL 7.1 program. Membranes were reblotted with anti-β-actin antibody as an internal control.

### Measurement of mRNA Expression

The expression of OPN mRNA in VSMCs was quantified by reverse transcription polymerase chain reaction (RT-PCR) using GAPDH as an internal standard. In brief, total RNA was isolated from cells using Qiazol (Qiagen, Hilden, Germany) and reverse transcribed into cDNA using the Improm-II Reverse Transcription System (Promega, Madison, WI, United States). cDNA amplification was performed using primers specific for OPN (forward, 5'-AGA CTG GCA GTG GTT TGC TT-3'; reverse, 5'-ATG GCT TTC ATT GGA GTT GC-3'). Equal amounts of RT-PCR products were separated on 1% agarose gel stained with ethidium bromide. Signal bands were quantified using the UNSCAN-IT GEL 7.1 program and data were expressed as relative GAPDH densities.

### Preparation of OPN Promoter Constructs and Luciferase Assay

As described in our previous report (Jang et al., 2017), a series of OPN promoter constructs in luciferase expression vector pGL3 basic (Promega) were prepared. The OPN promoter was amplified from genomic DNA using the following PCR primers (forward 5'-AGT GTA GGA AGC AGT CAG TCC TGT CAG-3'; reverse 5'-TAC CTT GGC TGG CTT CTC GAG CAT GCT-3') and then cloned into pGL3 basic to generate a pLuc-OPN-2284 construct. Additional deletion constructs lacking distal promoter sequences (denoted pLuc-OPN-538 and pLuc-OPN-234) were prepared by digesting pLuc-OPN-2284 with restriction enzymes (NheI, Sac1 or Xho1). All plasmids were prepared using the QIAprep spin kit (Qiagen Inc.). Cells were transfected with the plasmids using Lipofectamine 2000 Transfection Reagent (Invitrogen), according to the manufacturer’s instructions. Cell lysates were prepared using the passive lysis buffer from the Promega assay system, and luciferase activity was determined using the dual luciferase reporter assay system (Promega).

### Statistical Analysis

Results were expressed as means±SEMs. One-way ANOVA followed by Dunnett multiple comparison test or student’s *t*-test was used to determine significant differences. Statistical significance was accepted for values of *p*<0.05.

## Results

### Effect of HMGB1 on VSMC Migration

**Figure 1:**
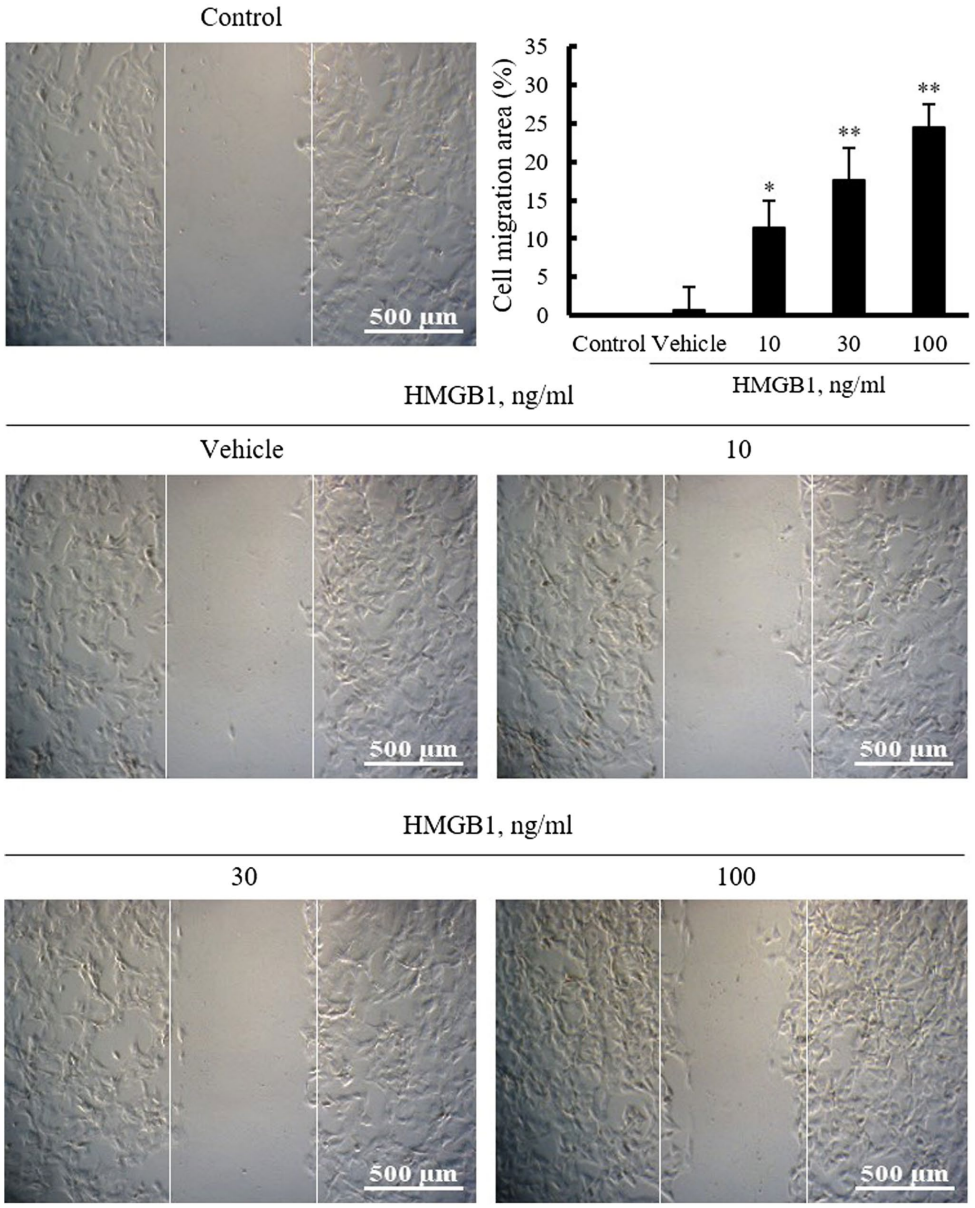
The effects of HMGB1 on VSMC migration. Rat aortic VSMCs were treated with HMGB1 (10 to 100ng/ml) for 48h. Cell migration was determined by wound-healing assay. Representative photomicrographs of cell monolayers at 48h post-wounding. Cell migration area (%) was calculated from the ratio of changes in wound area. Scale bar: 500μM. Data were quantified, expressed as the mean±SEM of six independent experiments. ^*^p<0.05 and ^**^p<0.01 vs. value in control.

To identify the pathogenic molecules involved in vascular remodeling in the injured vasculatures, this study investigated the role of HMGB1 on the migration of VSMCs cultured from rat thoracic aorta. In this study, VSMCs serum starved for 24h were wounded and then stimulated with HMGB1 (10–100ng/ml) for 48h. As shown in Figure 1, cell migration area was increased in a dose-dependent manner up to 100ng/ml concentration of HMGB1.

### Characteristics of OPN Expression in VSMCs Stimulated With HMGB1

**Figure 2:**
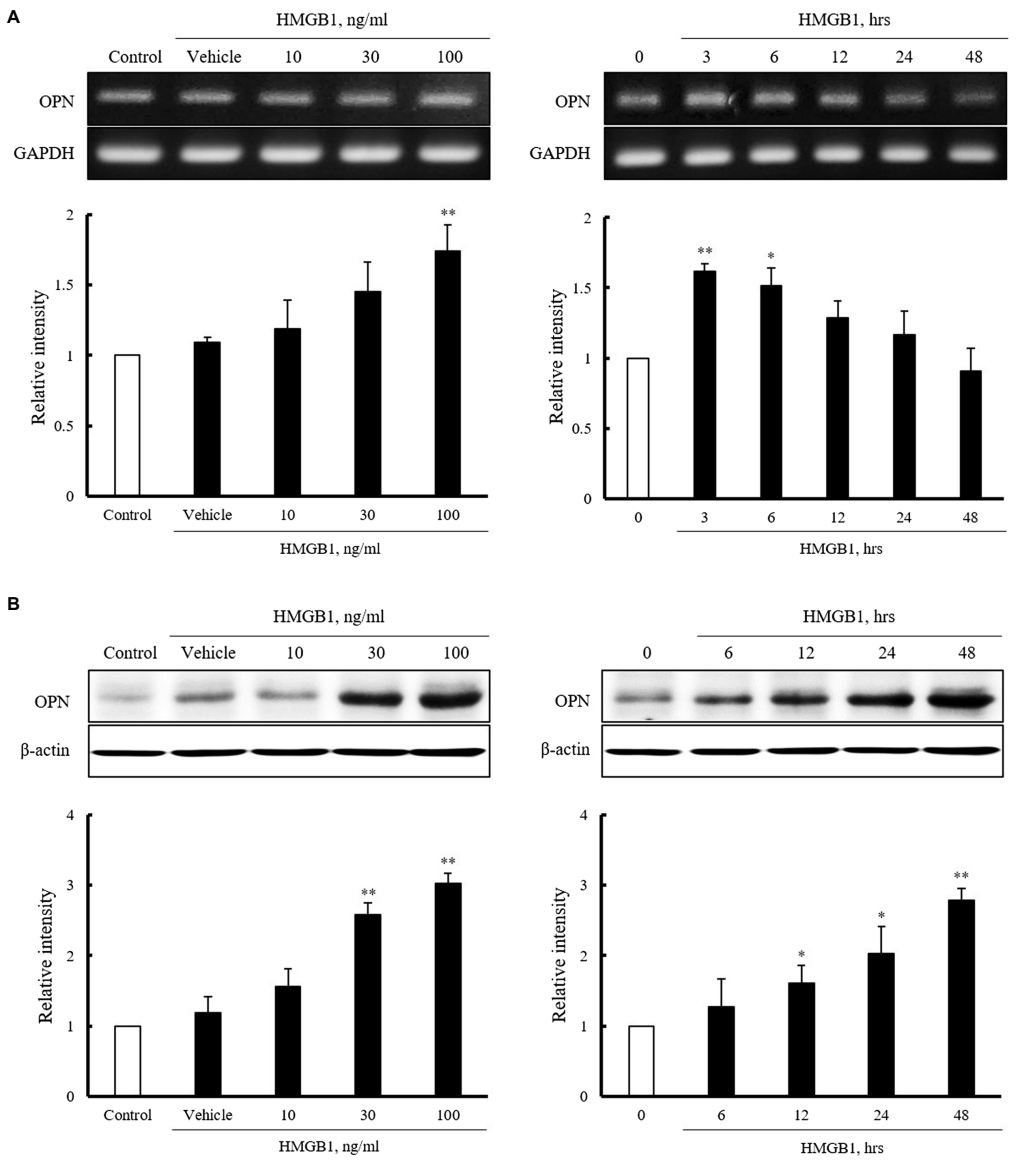
Characteristics of OPN mRNA and protein expression in VSMCs stimulated with HMGB1. **(A)** VSMCs were treated with HMGB1 (10 to 100ng/ml) for 3h or 100ng/ml of HMGB1 for 0 to 48h, and then, mRNA levels of OPN were determined by RT-PCR. GAPDH was used as a control. Relative intensities were expressed as the mean±SEM of 3–4 independent experiments. ^*^p<0.05 and ^**^p<0.01 vs. corresponding value in control or 0h. **(B)** VSMCs were stimulated with HMGB1 (10 to 100ng/ml) for 48h or 100ng/ml of HMGB1 for 0 to 48h, and then, protein levels of OPN were determined by Western blot. β-actin was used as a control. Relative intensities were expressed as the mean±SEM of 4–6 independent experiments. ^*^p<0.05 and ^**^p<0.01 vs. corresponding value in control or 0h.

Vascular smooth muscle cells were stimulated with HMGB1 (10 to 100ng/ml) for 3h or 100ng/ml of HMGB1 for 0 to 48h, and then, mRNA expression of OPN was determined by RT-PCR. As shown in Figure 2A, the expression of OPN mRNA in cells stimulated with HMGB1 was increased in a dose-dependent manner. At concentration of 100ng/ml of HMGB1, the cellular expression of OPN mRNA was peaked at 3h of HMGB1 stimulation and then gradually decreased until 48h. Likewise, in Western blot analysis, OPN protein expression was increased dose-dependently up to 100ng/ml HMGB1. The expression of OPN protein was time-dependently increased until 48h of 100ng/ml HMGB1 treatment (Figure 2B).

### Role of OPN in HMGB1-Induced VSMC Migration

**Figure 3:**
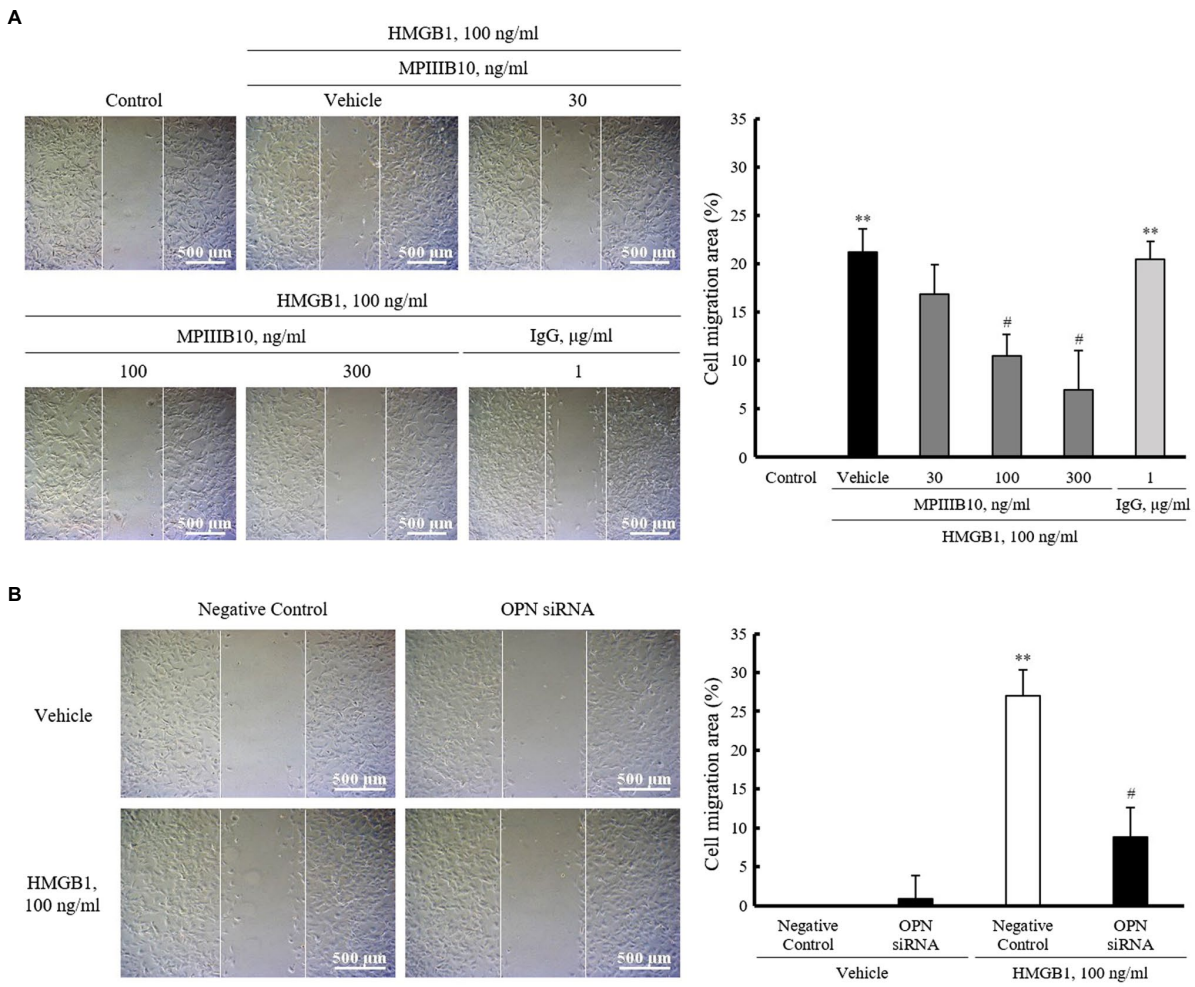
The role of OPN on HMGB1-induced VSMC migration. **(A)** VSMCs were pretreated with MPIIIB10 (30 to 300ng/ml) or IgG (1μg/ml) for 1h and then stimulated with HMGB1 (100ng/ml) for 48h. Cell migration was determined by wound-healing assay. Cell migration area (%) was calculated from the ratio of changes in wound area. Scale bar: 500μm. Data were quantified, expressed as the mean±SEM of five independent experiments. ^**^p<0.01 vs. value in control and ^#^*p*<0.05 vs. corresponding value in vehicle. **(B)** VSMCs were transfected with negative control siRNA or OPN siRNA (200nM) for 48h and then stimulated with HMGB1 (100ng/ml) for 48h. Cell migration was determined by wound-healing assay. Cell migration area (%) was calculated from the ratio of changes in wound area. Scale bar: 500μm. Data were quantified, expressed as the mean±SEM of four independent experiments. ^**^p<0.01 vs. value in negative control in vehicle and ^#^*p*<0.05 vs. corresponding value in negative control in HMGB1.

To evaluate the role of OPN in HMGB1-induced VSMC migration, VSMCs were pretreated with MPIIIB10 (30–300ng/ml), a neutralizing monoclonal antibody for OPN, and then, cell migration was induced by HMGB1. As shown in Figure 3A, the increased VSMC migration induced by 100ng/ml of HMGB1 for 48h was dose-dependently attenuated by pretreatment with MPIIIB10 at concentrations of 30 to 300ng/ml, but not by IgG at 1μg/ml concentration. Likewise, HMGB1-induced migration of VSMCs was markedly attenuated in cells transfected with 200nM of OPN siRNA (Figure 3B).

### Identification of the Transcription Factors Mediating OPN Expression in HMGB1-Stimulated VSMCs

**Figure 4:**
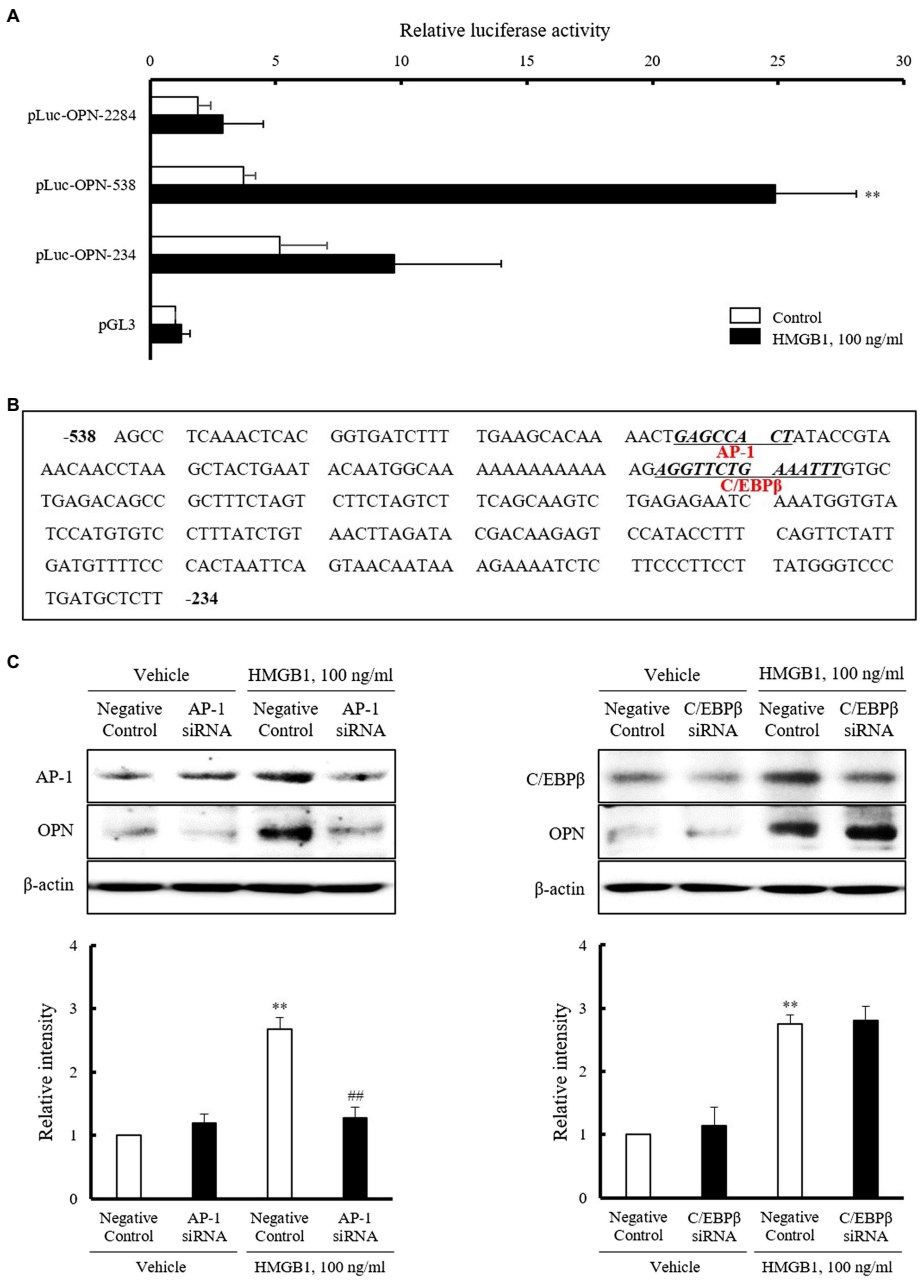
Identification of the transcription factors mediating OPN expression in HMGB1-stimulated VSMCs. **(A)** VSMCs were transfected with various promoter constructs or an empty luciferase vector (pGL3) for 48h and then stimulated with HMGB1 (100ng/ml) for 3h. Relative luciferase activities were expressed as the mean±SEM of four independent experiments. ^**^p<0.01 vs. corresponding value in control. **(B)** Nucleotide sequence of the –538~–234 promoter region of the OPN gene is shown. The sequence of potential binding sites for AP-1 and C/EBPβ in pLuc-OPN-538 were underlined. The transcription factor-binding sites were identified using TF search software. **(C)** VSMCs were transfected with AP-1 and C/EBPβ siRNAs (200nM) for 48h and then stimulated with HMGB1 (100ng/ml) for 48h. The expression of OPN was determined by Western blot. β-actin was used as a control. Relative intensities were expressed as the mean±SEM of five independent experiments. ^**^p<0.01 vs. corresponding value in negative control in vehicle and ^##^*p*<0.01 vs. value in negative control in HMGB1.

To identify the regions responsible for HMGB1-induced OPN transcription within 2284 region promoter, luciferase activity was measured after transient transfection of three constructs with different promoter sizes (pLuc-OPN-2284, –538, and –234) into VSMCs. As shown in Figure 4A, the luciferase reporter activity of pLuc-OPN-538 in VSMCs exposed to 100ng/ml HMGB1 for 3h was about 6.7(±3.68)-fold higher than that in control. This increase in HMGB1-induced luciferase activity was abolished in cell transfected with the pLuc-OPN-234 construct. These results suggest that the –538~–234 region of the OPN promoter is responsible for HMGB1-induced OPN transcription in VSMCs. Putative-binding sites for AP-1 and C/EBPβ in this region were suggested by a TF Search program (Figure 4B). As shown in Figure 4C, the increased binding of AP-1 and C/EBPβ in HMGB1-treated VSMCs was demonstrated. The HMGB1-induced OPN expression was reduced in cells transfected with AP-1 siRNA, but not in cells transfected with C/EBPβ siRNA, indicating a pivotal role for AP-1 in the transcriptional regulation of OPN in VSMCs stimulated with HMGB1.

### Role of AP-1 in HMGB1-Induced VSMC Migration

**Figure 5:**
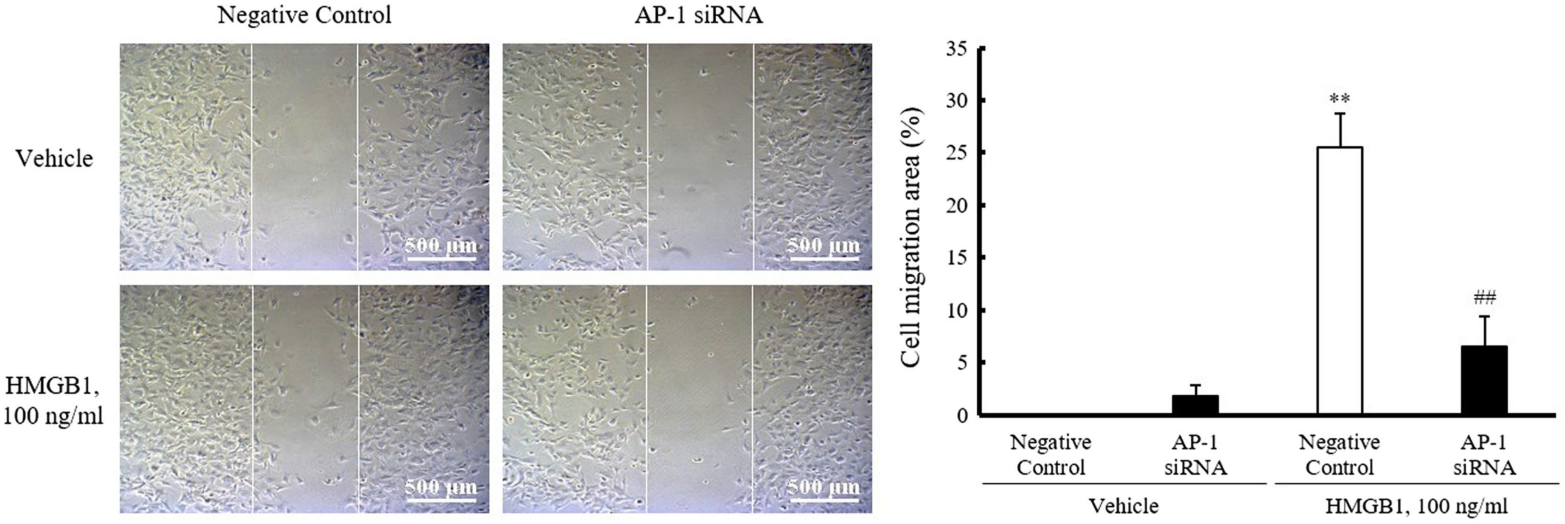
Role of AP-1 in HMGB1-induced VSMC migration. VSMCs were transfected with AP-1 siRNA (200nM) for 48h and then stimulated with HMGB1 (100ng/ml) 48h. Cell migration was determined by wound-healing assay. Representative photomicrographs of cell monolayers at 48h post-wounding. Cell migration area (%) was calculated from the ratio of changes in wound area. Scale bar: 500μm. Data were quantified, expressed as the mean±SEM of five independent experiments. ^**^p<0.01 vs. value in negative control in vehicle and ^##^*p*<0.01 vs. corresponding value in negative control in HMGB1.

To investigate the role of AP-1 on HMGB1-induced VSMC migration, cells were wounded after transfection with AP-1 siRNA (200nM) and then stimulated with 100ng/ml of HMGB1. As shown in Figure 5, the migration of negative control-transfected cells was markedly increased in cells stimulated with 100ng/ml HMGB1 for 48h. However, HMGB1-incuced cell migration was significantly attenuated in cells transfected with AP-1 siRNA, suggesting an essential role of AP-1-mediated OPN expression in VSMC migration in the injured vasculatures.

## Discussion

Increased migration of VSMCs is a pivotal pathogenic event mediating vascular remodeling diseases, such as atherosclerosis and vascular restenosis in the injured vasculatures (Li et al., 2007). In this study, VSMC migration was increased in response to HMGB1, a major DAMP implicated in vascular inflammation. HMGB1-induced migration of VSMCs was attenuated in cells deficient of OPN as well as in cells pretreated with MPIIIB10, a neutralizing monoclonal antibody for OPN. Moreover, increased bindings between AP-1 and OPN promoter in VSMCs stimulated with HMGB1 were demonstrated, suggesting an important role for AP-1-mediated OPN expression in the increased migration of HMGB1-stimulated VSMCs.

Damage-associated molecular patterns have been implicated in the progression of several metabolic diseases, such as obesity, type 2 diabetes, and atherosclerosis (Garcia-Martinez et al., 2015). Thus, the modulation of altered cellular functions induced by DAMPs might be important in preventing or treating various vascular diseases. Among the various injury-induced mediators, HMGB1 is one of the best characterized DAMPs in the development and progression of cardiovascular diseases (Land, 2013; Cai et al., 2015; Nishibori et al., 2019). An increased level of HMGB1 was demonstrated in atherosclerotic plaque and suggested to be involved in vascular remodeling *via* the potentiation of inflammatory processes (Kalinina et al., 2004; Cai et al., 2015). Also, recent evidences have indicated that HMGB1 is required for the development of neointimal lesions following vascular injury (Chen et al., 2012; Zou et al., 2014). Although VSMC migration plays a pivotal role in pathogenesis of vascular remodeling, the precise role of HMGB1 on VSMC migration is unclear. To investigate the effects of HMGB1 on VSMC migration, VSMCs cultured from rat thoracic aorta were wounded and then stimulated with HMGB1. In our present study, VSMC migration induced by HMGB1 was increased in a dose-dependent manner. Thus, HMGB1 released in the injured vasculatures was suggested to be a key player in the development and progression of vascular remodeling *via* an increased migration of VSMCs.

Osteopontin is expressed in a wide range of cell types including some epithelia, macrophages, and VSMCs in a constitutive and inducible fashion (Brown et al., 1992). Reportedly, OPN increases the migration (Liaw et al., 1995) and proliferation (Parrish and Ramos, 1997) of VSMC, production of matrix metalloproteinases (Isoda et al., 2002), vascular calcification (Giachelli et al., 1993), and also involved in vascular inflammation (Mohamadpour et al., 2015). In line with previous other reports, our previous studies demonstrated that OPN expression was increased in VSMCs stimulated with PDGF and 4-hydroxynonenal and mediated VSMC proliferation induced by these two stimulants (Lee et al., 2016; Jang et al., 2017). As intracellular signals mediating OPN-induced cell migration, p38 MAPK signaling pathway was suggested to mediate OPN-promoted cell migration and invasion in colorectal cancer cells (Huang et al., 2017). In VSMCs, OPN stimulated VSMC migration by inducing focal adhesion kinase phosphorylation and integrin-linked kinase dephosphorylation (Li et al., 2007). Based on these reports, OPN has emerged as a key factor in vascular remodeling diseases, such as atherosclerosis and vascular restenosis.

To determine the importance of OPN in VSMC migration in the injured vasculatures, VSMCs were exposed to HMGB1 and then determined OPN expression in VSMCs, and also evaluated the role of OPN signaling in HMGB1-induced VSMC migration. Our present study shows that OPN mRNA and protein levels were markedly increased in VSMCs stimulated with HMGB1. Using the wound-healing assay, it was demonstrated that HMGB1-induced VSMC migration was significantly attenuated in cells pretreated with MPIIIB10, a neutralizing monoclonal antibody for OPN as well as in cells deficient of OPN. Based on these results, it was indicated that OPN played a pivotal role in VSMC migration induced by HMGB1. Considering the importance of focal adhesion kinase and integrin-linked kinase in OPN-induced VSMC migration (Li et al., 2007), these molecules might be suggested as potential intracellular signals mediating VSMC migration induced by HMGB1. The potential importance of these signal pathways in OPN-induced migration of VSMCs in the injured vasculatures needs to be clarified.

Cell migration *in vivo* or *in vitro* begins with stimulation of cell surface receptors that transduce the external signal to a series of coordinated remodeling events that alter the structure of the cytoskeleton. Therefore, clarifying the signal-transduction pathways and their effects on cell biological behaviors are helpful in understanding the molecular mechanisms of those remodeling cardiovascular diseases. Among OPN-activated cell surface receptors including CD44 and integrin receptors, αvβ3 integrin signaling was demonstrated to be involved in the OPN-induced VSMC migration in the injured vasculatures (Yue et al., 1994). Thus, the involvement of integrin receptors in OPN-induced migration of HMGB1-stimulated VSMCs was suggested; however, further experiments are needed to determine the individual role of cell surface receptors mediating OPN-induced VSMC migration in the injured vasculatures.

Results of our present study provide an important insight of the mechanism responsible for HMGB1-enhanced OPN expression in primary cultured VSMC. In the present study, HMGB1-induced OPN expression was found to be regulated at the transcriptional levels. The luciferase reporter activity of pLuc-OPN-538 in VSMCs exposed to 100ng/ml of HMGB1 was about 6.7(±3.68)-fold higher than the control, whereas this increase was not observed in cells transfected with the pLuc-OPN-234 and –2284 constructs. These results suggest that the –538~–234 region of OPN promoter is the cis-acting element responsible for HMGB1-induced OPN transcription in VSMC. Using the sequence motif search of TF Search software,^1^ putative transcription factor-binding sites for AP-1 and C/EBPβ were identified between –538bp and –234bp relative to the transcriptional initiation site in the OPN promoter.

The pivotal role for AP-1 complex proteins c-Fos and c-Jun in VSMC proliferation induced by HMGB1 was reported in the previous study (Zabini et al., 2015); however, the role of AP-1 in VSMC migration is poorly understood. To further investigate the role of AP-1 and C/EBPβ signaling in HMGB1-induced OPN expression, OPN expression was determined using AP-1- and C/EBPβ-deficient cells stimulated with HMGB1. In response to HMGB1, OPN expression was markedly attenuated in AP-1-deficient VSMCs, but not in C/EBPβ-deficient cells. Thus, it was suggested that the transcription factor AP-1 was essential for HMGB1-induced OPN transcription in VSMCs. In addition, this study provides direct *in vitro* evidence that the exposure of VSMC to HMGB1 increases the expression of AP-1, leading to higher expression of OPN, in association with an increased migration of VSMCs. The increased migration of VSMC induced by HMGB1 was markedly attenuated in cells deficient of AP-1 using AP-1 siRNA, suggesting a pivotal role of HMGB1-AP-1-OPN axis in VSMC migration. However, further *in vivo* experiment using HMGB1-deficient mice is necessary to support our suggested conclusion based on only *in vitro* data.

Taken together, our data suggest that HMGB1-induced VSMC migration through an increased expression of OPN *via* AP-1 signaling pathway. Thus, the HMGB1-AP-1-OPN signaling axis in VSMCs might serve as a potential therapeutic target for vascular remodeling in the injured vasculatures.

## Data Availability Statement

The original contributions presented in the study are included in the article/supplementary material, further inquiries can be directed to the corresponding author.

## Ethics Statement

The animal study was reviewed and approved by Pusan National University Institutional Animal Care and Use Committee.

## Author Contributions

EYJ and SEB designed and performed the experiments, analyzed the experimental data, and wrote the manuscript. CDK contributed to design and the writing. JOK, JMC, and EJJ performed the experiments. All authors approved the final manuscript.

## Funding

This research was supported by the Basic Science Research Program (NRF-2020R1A2C1005135) and the Medical Research Center (MRC) Program (NRF-2015R1A5A2009656) through the National Research Foundation of Korea (NRF) grant funded by the Korean government (MSIP).

## Conflict of Interest

The authors declare that the research was conducted in the absence of any commercial or financial relationships that could be construed as a potential conflict of interest.

The reviewer SEB declared a shared affiliation with several of the authors (CDK, EYJ, SEB, JOK, JMC, and EJJ), to the handling editor at time of review.

## Publisher’s Note

All claims expressed in this article are solely those of the authors and do not necessarily represent those of their affiliated organizations, or those of the publisher, the editors and the reviewers. Any product that may be evaluated in this article, or claim that may be made by its manufacturer, is not guaranteed or endorsed by the publisher.

## Footnotes

^1^ http://mbs.cbrc.jp/research/db/TFSEARCH.html
